# Association between glycemic variability and acute kidney injury incidence in patients with cerebral infarction: an analysis of the MIMIC-IV database

**DOI:** 10.3389/fendo.2025.1615051

**Published:** 2025-06-12

**Authors:** Yiming Hua, Ze Chen, Lele Cheng, Ning Ding, Yifei Xie, Hao Wu, Huaizhi Jing, Yu Xu, Yue Wu, Beidi Lan

**Affiliations:** ^1^ Department of Cardiovascular Medicine, The First Affiliated Hospital of Xi’an Jiaotong University, Xi’an, Shaanxi, China; ^2^ Key Laboratory of Molecular Cardiology, Key Laboratory of Environment, Genes Related to Diseases, Ministry of Education Xi’an Jiaotong University, Xi’an, Shaanxi, China; ^3^ Department of Vascular Surgery, The First Affiliated Hospital of Xi’an Jiaotong University, Xi’an, China; ^4^ Department of Medical Imaging, The First Affiliated Hospital of Xi’an Jiaotong University, Xi’an, China; ^5^ Department of Cardiovascular Surgery, The First Affiliated Hospital of Xi’an Jiaotong University, Xi’an, China

**Keywords:** glycemic variability, cerebral infarction, acute kidney injury, MIMIC-IV, ICU

## Abstract

**Introduction:**

Glycemic variability (GV) is an increasingly important predictive indicator of vascular occlusion-related complications. Studies have demonstrated that a higher GV is associated with poor outcomes in patients with cerebral infarction (CI). The prognostic utility of GV in CI patients for predicting acute kidney injury (AKI) remains inadequately characterized. This investigation systematically examines the pathophysiological relationship between acute glycemic fluctuations and AKI development in CI populations, with particular emphasis on temporal patterns of glucose dysregulation.

**Methods:**

This retrospective cohort analysis utilized data from the MIMIC-IV database, categorizing CI patients into quartiles based on GV metrics. Primary outcomes included AKI incidence and renal replacement therapy (RRT) initiation, with in-hospital mortality designated as the secondary endpoint. Analytical methodologies employed Kaplan-Meier survival curves with log-rank testing, multivariable-adjusted Cox proportional hazards regression, and logistic regression modeling to evaluate GV-AKI associations while controlling for critical confounders.

**Results:**

The analytical cohort comprised 3,343 critically ill individuals extracted from the MIMIC-IV database. Kaplan-Meier curve analysis demonstrated progressively elevated cumulative risks of AKI development, RRT requirement, and in-hospital mortality among individuals with heightened GV. Following multivariable adjustment, logistic regression models and Cox proportional hazards analyses confirmed GV as an independent predictor of AKI progression, RRT dependency, and mortality risk in cerebral infarction patients.

**Conclusion:**

This investigation identifies GV as an independent prognostic determinant for AKI development in cerebral infarction patients. GV demonstrates clinical utility as a biomarker for stratifying AKI risk in this population.

## Introduction

CI is a serious neurological disorder, which is the main cause of disability and mortality worldwide. The brain and kidneys have similar physiological characteristics in terms of anatomy, vascular regulation, and hemodynamics. Through central/autonomic pathways and immune interactions, the neurorenal axis normally sustains systemic homeostasis, whose dysfunction - as seen in hypertensive sympathoexcitation - disrupts pressure-natriuresis mechanisms (1). Studies have shown that approximately 11.60% of patients with ischemic stroke have AKI, which increases the risk of poor prognoses (2). The prevalence of dialysis-requiring AKI among cerebrovascular accident inpatients demonstrates a progressive upward trajectory, exhibiting robust correlations with elevated mortality risk and non-routine discharge dispositions (3). Although accumulating evidence has shown an association between CI and renal dysfunction, the underlying mechanisms remain unclear. Early identification and targeted mitigation of AKI risk determinants post-CI are essential for optimizing prognostic trajectories.

GV represents a composite biomarker quantifying both acute fluctuations and chronic alterations in blood glucose (BG) homeostasis, serving as a dynamic indicator of glucoregulatory efficacy (4). This pathophysiological mechanism is orchestrated through multisystemic interactions involving the neuroendocrine axis and peripheral organ systems. Clinical evidence identifies infectious processes, surgical interventions, traumatic injuries, critical comorbidities, and pharmacological agents as principal etiological drivers of stress-induced glycemic dysregulation in ICU settings (5). Research has shown that patients with severe hyperglycemia require longer hospital stays and exhibit higher invasive ventilation and mortality rates; however, imatinib significantly lowers the incidence of severe hyperglycemia (6). Weekly subcutaneous semaglutide administration demonstrated superior efficacy to placebo in reducing cardiovascular mortality and nonfatal cardiovascular events (myocardial infarction and stroke) over a mean 39.8-month follow-up period (7). Interestingly, the lower the BG control, the less likely it is to increase the incidence of long-term benign events. Ma et al. reported that hypoglycemia was strongly associated with ICU mortality among patients without diabetes mellitus (DM) and less so among those with DM (8). Recently, GV, which is a comprehensive evaluation of hyperglycemia and hypoglycemia, has become a significant indicator of glycemic control. In contrast to static glycemic parameters (mean BG), GV demonstrates superior clinical utility as a dynamic biomarker of glucose fluctuation magnitude. Among individuals with diabetes mellitus, heightened GV independently predicts elevated risks of major adverse limb events, including peripheral arterial disease progression and critical limb ischemia development (9). Elevated GV independently predicts heightened 90-day mortality risk and represents a critical modifiable prognostic determinant in critically ill patients with AKI (10). Elevated glycemic variability (GV), indicative of impaired blood glucose homeostasis, exhibits a dose-dependent association with heightened risks of major adverse cardiocerebrovascular and renal events (MACCRE). Nevertheless, the pathophysiological interplay between GV and AKI development in CI populations remains inadequately characterized.Specifically, CI patients often present with stress-induced hyperglycemia, autonomic dysfunction, and a high prevalence of comorbidities such as hypertension and diabetes, all of which may predispose them to acute kidney injury (AKI). Additionally, blood glucose variability in CI patients is more pronounced due to the neuroendocrine disturbances associated with acute cerebral events. Therefore, investigating GV in this specific population may reveal clinically relevant insights that are not as apparent in the general population.

This investigation employed Kaplan-Meier survival analysis to delineate the temporal relationship between GV and AKI development in CI populations. To validate these associations, we applied multivariate-adjusted logistic regression and Cox proportional hazards models, complemented by stratified subgroup evaluations to systematically investigate the interdependent associations between RRT utilization, in-hospital mortality, and CI-AKI progression.

## Methods

### Data source and study population

**Figure 1:**
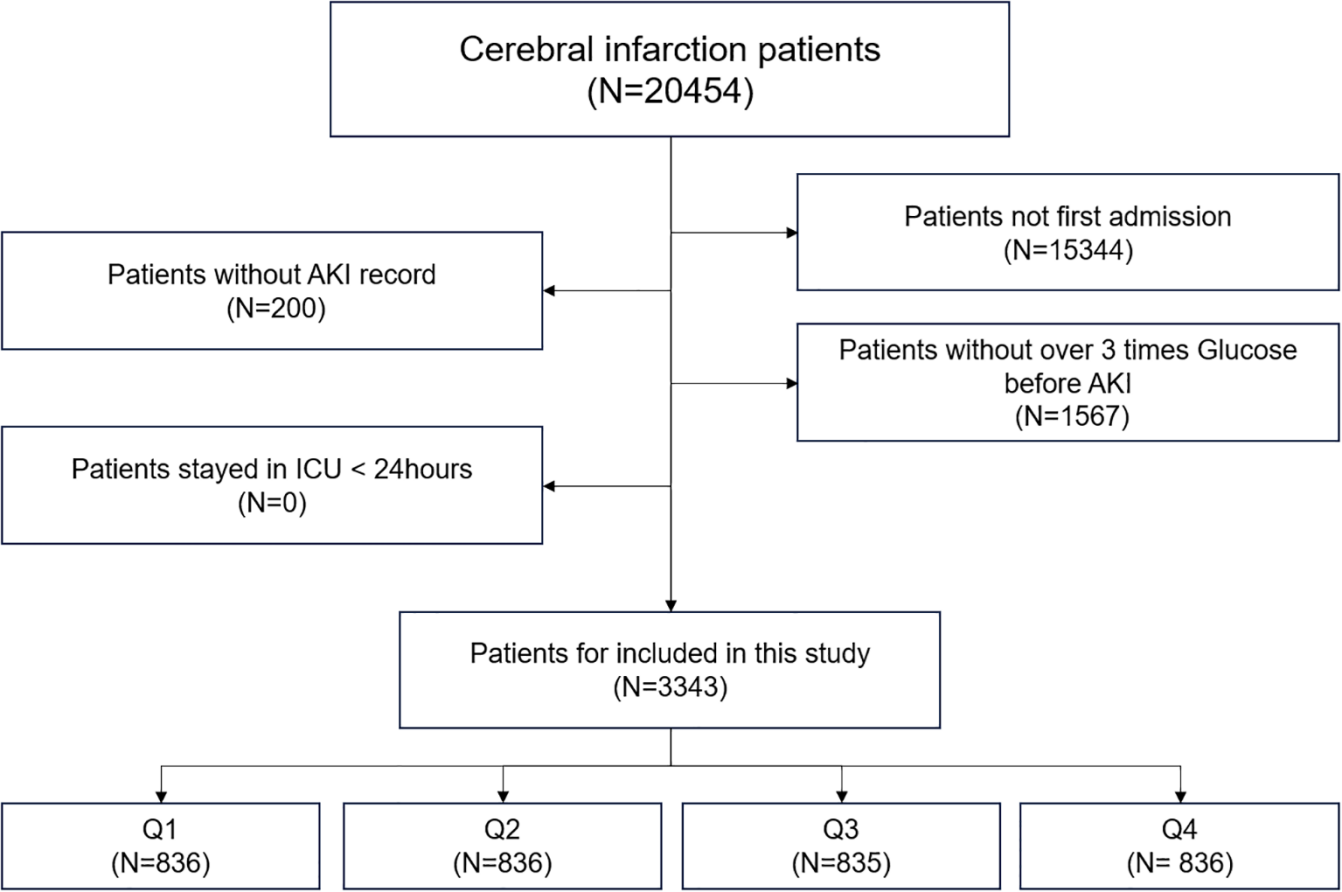
Flowchart of extraction of patients with cerebral infarction from Medical Information Mart for the Intensive Care-IV database for this study.

We retrospectively retrieved data on patients with sepsis from the Medical Information Mart for the Intensive Care (MIMIC)-IV database, which includes comprehensive information on all patients treated at the Beth Israel Deaconess Medical Center in Boston, Massachusetts, between 2008 and 2019. The data included vital signs, laboratory test results, medications, risk assessment indicators, and other relevant information. The requirement for research qualifications was waived (official certification number: 52681986 for the first author Yiming Hua). Owing to demands for privacy protection, databases de-identify personal information by replacing patients identifiers with random codes. CI is a type of stroke that occurs when a blood vessel supplying blood to the brain is obstructed, leading to a lack of oxygen and nutrients in the affected area of the brain. The deprivation of blood flow results in brain cell death and subsequent neurological deficits (11). The exclusion criteria for the MIMIC-IV database were as follows: (1) not being admitted for the first time, (2) not having AKI records, (3) not having more than three glucose tests before AKI, and (4) ICU stay <24 h (**Figure 1**).

### Data extraction

In this study, we used a Structured Query Language to extract data from the Navicat Premium software (version 12.0). To quantify GV, we derived the coefficient of variation (CV) using the formula CV = [standard deviation (SD) of blood glucose/MBG] × 100%, which provides a normalized measure of glucose fluctuation independent of absolute glucose levels. All measurements were obtained before the occurrence of AKI. All data obtained from the MIMIC-IV database within 24 h of admission included age; sex; white blood cell (WBC), red blood cell (RBC), and platelet counts; hemoglobin (HB), blood urea nitrogen (BUN), serum creatinine (SCR), alanine aminotransferase (ALT), aspartate aminotransferase (AST), sodium, potassium, calcium, and chloride levels; hypertension (HTN); chronic heart failure (CHF); presence of chronic obstructive pulmonary disease (COPD), DM, chronic kidney disease (CKD), and AKI; RRT use; and in-hospital mortality. The follow-up period began on the first day of hospitalization and ended at the onset of AKI. The secondary endpoint extended from the initial admission to the first use of RRT or death during the follow-up period.

### Primary and secondary outcomes

The primary endpoint of this study was the incidence of AKI. The diagnostic criteria for AKI were based on the Kidney Disease: Improving Global Outcomes (KDIGO) guidelines, which define AKI as either an increase in serum creatinine (SCR) to ≥1.5 times the baseline within 7 days, an increase of ≥0.3 mg/dL in SCR within 24 hours, or oliguria (12). The baseline SCR level used in this study was that measured within the first 24 h of admission. RRT is an indirect indicator of AKI severity and was considered a secondary endpoint.

### Statistical analysis

Continuous variables were presented as means and standard deviations and compared between groups using either the Mann–Whitney U test or Student’s t-test. Categorical variables were expressed as frequencies and percentages and compared between groups using Fisher’s exact test or Pearson’s chi-square test.

Kaplan–Meier survival analysis was used to assess the correlation between GV and AKI incidence in each group. Additionally, Cox proportional hazards model analysis was performed to calculate the hazard ratios (HRs) and their corresponding 95% confidence intervals (CIs) for the impact of GV on AKI incidence across different groups. The analysis adjusted for multiple variables that may have influenced the outcomes. No variable adjustments were made in model 1. Model 2 was adjusted for sex and age. As illustrated in **Table 1**, the following variables were included in model 3: age; sex; WBC and RBC counts; HB, SCR, BUN, ALT, AST, sodium, calcium, and chloride levels; and presence of CHF, HTN, DM, and CKD. Each model incorporated GV in both continuous and categorical forms. Q1 served as the baseline group in all models. Furthermore, restricted cubic splines were applied to examine the relationship between GV and AKI incidence based on the fully adjusted model 3. We also employed competing risk analysis, specifically the Fine-Gray model, to account for the confounding effect of in-hospital mortality on the incidence of AKI and RRT, thereby minimizing bias introduced by competing events.

**Table 1:** Cox regression model for the association of glycemic variability with acute kidney injury incidence and renal replacement therapy use and logistic regression model for its association with in-hospital mortality in the Medical Information Mart for the Intensive Care database.

Categories	Model 1		Model 2		Model 3	
	HR (95%CI)	Pvalue	HR (95%CI)	Pvalue	HR (95%CI)	Pvalue
AKI incidence						
GV as continuous	1.788 [95%CI 1.422-2.248]	<0.001	1.776 [95%CI 1.411-2.237]	<0.001	1.349 [95%CI 0.970-1.877]	0.076
Quartile^a^						
Q1	Ref.		Ref.		Ref.	
Q2	1.199 [95%CI 1.073-1.339]	0.001	1.186 [95%CI 1.061-1.325]	0.003	1.166 [95%CI 1.007-1.350]	0.040
Q3	1.306 [95%CI 1.170-1.458]	<0.001	1.303 [95%CI 1.166-1.456]	<0.001	1.299 [95%CI 1.121-1.505]	<0.001
Q4	1.446 [95%CI 1.296-1.612]	<0.001	1.439 [95%CI 1.289-1.606]	<0.001	1.320 [95%CI 1.133-1.537]	<0.001
RRT incidence						
GV as continuous	8.064 [95%CI 4.921-13.220]	<0.001	9.915 [95%CI 6.020-16.330]	<0.001	4.539 [95%CI 1.753-11.752]	0.002
Quartile^a^						
Q1	Ref.		Ref.		Ref.	
Q2	4.186 [95%CI 1.934-9.063]	<0.001	4.384 [95%CI 2.025-9.494]	<0.001	2.222 [95%CI 0.883-5.590]	0.090
Q3	5.900 [95%CI 2.785-12.501]	<0.001	6.531 [95%CI 3.080-13.850]	<0.001	2.609 [95%CI 1.065-6.392]	0.036
Q4	11.424 [95%CI 5.538-23.567]	<0.001	13.362 [95%CI 6.459-27.646]	<0.001	4.285 [95%CI 1.789-10.261]	0.001
In-hospital mortality						
GV as continuous	1.429 [95%CI 1.316-1.551]	<0.001	1.421 [95%CI 1.309-1.543]	<0.001	1.326 [95%CI 1.176-1.494]	<0.001
Quartile^a^						
Q1	Ref.		Ref.		Ref.	
Q2	1.068 [95%CI 1.034-1.103]	<0.001	1.066 [95%CI 1.032-1.101]	<0.001	1.065 [95%CI 1.018-1.114]	0.006
Q3	1.077 [95%CI 1.043-1.113]	<0.001	1.075 [95%CI 1.040-1.110]	<0.001	1.054 [95%CI 1.006-1.103]	0.025
Q4	1.161 [95%CI 1.124-1.200]	<0.001	1.158 [95%CI 1.121-1.196]	<0.001	1.135 [95%CI 1.082-1.191]	<0.001

Model 1 was unadjusted.

Model 2 was adjusted for sex and age.

Model 3 was adjusted for age; sex; white blood cell and red blood cell counts; hemoglobin, serum creatinine, blood urea nitrogen, alanine aminotransferase, aspartate aminotransferase, sodium, calcium, and chloride levels; and the presence of chronic heart failure, hypertension, diabetes, and chronic kidney disease.

The lowercase letter “a” following “quartile” indicates that, according to the research method described earlier, the two groups were divided based on the 25% quartile.

Subgroup analyses were conducted to explore the impact of the GV value on outcome indicators within various subgroups defined based on sex (female vs. male), age (<65 vs. ≥65 years) and presence of conditions, such as HTN, CHF, COPD, CKD and DM. All data analyses were performed using R version 4.2.3. Statistical significance was defined as a two-sided p-value < 0.05.

## Results

### Baseline characteristics

The basic characteristics of the patients with sepsis from the MIMIC-IV database are presented in **Table 2**. Based on their GV, patients are categorized into Q1 (0, 0.11), Q2 (0.11, 0.167), Q3 (0.167, 0.248), and Q4 (0.248, 1.88); the average GV for these groups were 0.08 ± 0.02, 0.14 ± 0.02, 0.20 ± 0.02, 0.38 ± 0.16. More importantly, AKI incidence was significantly higher in Q4 (84.2%) than in Q1 (71.2%), Q2 (78.1%), and Q3 (80.7%). More importantly, RRT use (10.4%) and in-hospital mortality (21.3%) were also significantly higher in Q4 than in the other three groups. In terms of demographic data, Q4 included older patients and a higher proportion of female patients, compared with the other three groups. Further, Q4 was characterized by higher WBC count and BUN, SCR, ALT, and AST levels but lower RBC count and HB, sodium, calcium, and chloride levels, compared with the other three groups. Additionally, the incidences of CHF, COPD, DM, and CKD were significantly higher, whereas the occurrence of HTN was significantly lower in Q4 than in the other three groups (all p<0.05).

**Table 2:** Baseline characteristics of patients according to GV value.

Categories	Q1 (0,0.11]	Q2 (0.11,0.167]	Q3 (0.167,0.248]	Q4 (0.248,1.88]	Pvalue
	(N=836)	(N=836)	(N=835)	(N=836)	
Demographic					
Age	69.44 ± 15.62	69.60 ± 14.49	71.30 ± 13.83	71.52 ± 13.99	0.003
Gender					0.011
Male	454 ( 54.3)	475 ( 56.8)	431 ( 51.6)	411 ( 49.2)	
Female	382 ( 45.7)	361 ( 43.2)	404 ( 48.4)	425 ( 50.8)	
Laboratory tests					
WBC (K/uL)	9.45 ± 4.30	10.10 ± 4.38	10.58 ± 8.83	10.70 ± 5.51	<0.001
RBC (K/uL)	4.08 ± 0.71	3.94 ± 0.79	3.93 ± 0.76	3.88 ± 0.79	<0.001
Platelets (K/uL)	235.40 ± 85.40	229.20 ± 107.51	230.59 ± 94.94	235.17 ± 103.03	0.461
Hemoglobin (g/dL)	11.62 ± 2.29	11.03 ± 2.42	10.77 ± 2.36	10.35 ± 2.37	<0.001
BUN (mg/dL)	17.05 ± 11.15	19.92 ± 14.71	21.55 ± 16.88	25.81 ± 20.27	<0.001
SCR (mg/dL)	0.94 ± 0.54	1.12 ± 1.03	1.16 ± 1.00	1.38 ± 1.46	<0.001
ALT (U/L)	27.66 ± 36.35	58.54 ± 257.27	64.53 ± 207.42	78.11 ± 250.20	<0.001
AST (U/L)	38.73 ± 129.34	78.70 ± 324.51	93.88 ± 302.95	99.73 ± 300.13	<0.001
Sodium (mEq/L)	138.01 ± 3.88	137.96 ± 4.51	137.56 ± 4.81	137.47 ± 5.10	0.031
Potassium (mEq/L)	3.89 ± 0.45	3.90 ± 0.50	3.93 ± 0.55	3.90 ± 0.56	0.560
Calcium (mg/dL)	8.55 ± 0.73	8.42 ± 0.80	8.37 ± 0.86	8.27 ± 0.90	<0.001
Chloride (mg/dlL)	103.03 ± 4.38	103.12 ± 5.28	102.68 ± 5.66	102.24 ± 6.28	0.008
GV	0.08 ± 0.02	0.14 ± 0.02	0.20 ± 0.02	0.38 ± 0.16	<0.001
Comorbidities, n(%)					
Hypertension	470 ( 56.2)	438 ( 52.4)	412 ( 49.3)	406 ( 48.6)	0.007
CHF	158 ( 18.9)	198 ( 23.7)	228 ( 27.3)	300 ( 35.9)	<0.001
COPD	135 ( 16.1)	153 ( 18.3)	165 ( 19.8)	204 ( 24.4)	<0.001
Diabetes	179 ( 21.4)	209 ( 25.0)	272 ( 32.6)	495 ( 59.2)	<0.001
CKD	126 ( 15.1)	136 ( 16.3)	184 ( 22.0)	240 ( 28.7)	<0.001
Events, n(%)					
AKI	595 ( 71.2)	653 ( 78.1)	674 ( 80.7)	704 ( 84.2)	<0.001
RRT	8 ( 1.0)	33 ( 3.9)	46 ( 5.5)	87 ( 10.4)	<0.001
Death at hospital	53 ( 6.3)	108 ( 12.9)	115 ( 13.8)	178 ( 21.3)	<0.001

### Association between GV and AKI incidence

**Figure 2:**
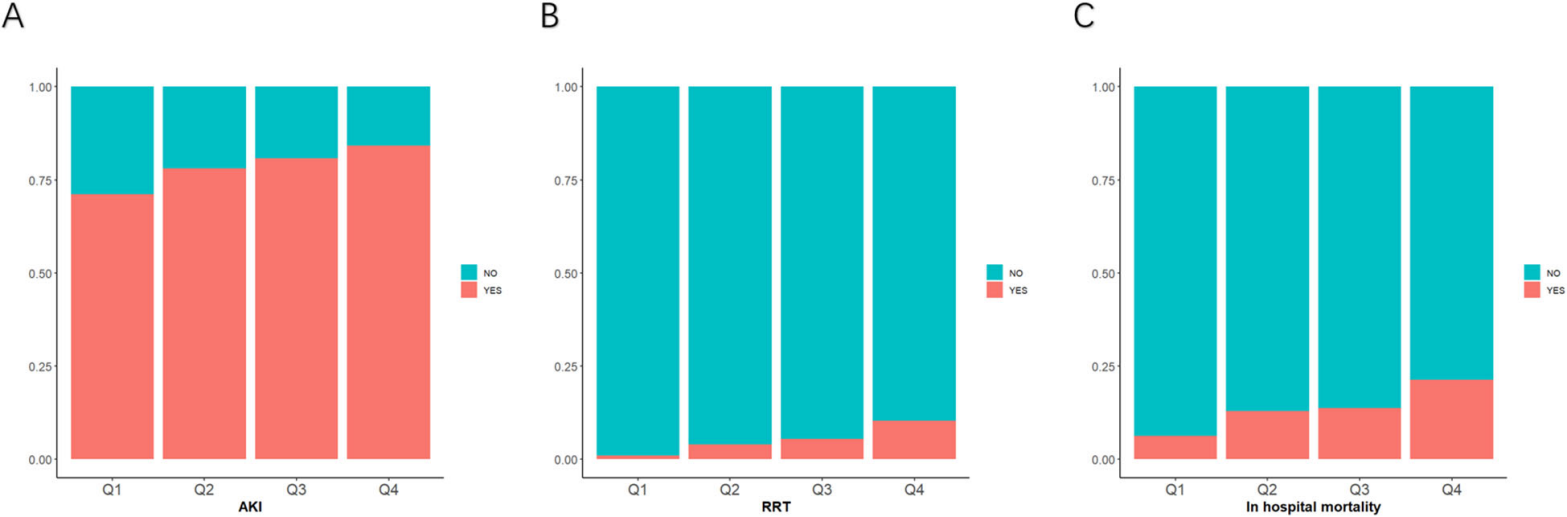
Stacked percentage bar chart for acute kidney injury (AKI) incidence, renal replacement therapy (RRT) use, and in-hospital mortality. **(A–C)** AKI incidence, RRT use, and in-hospital mortality are shown separately.

**Figure 3:**
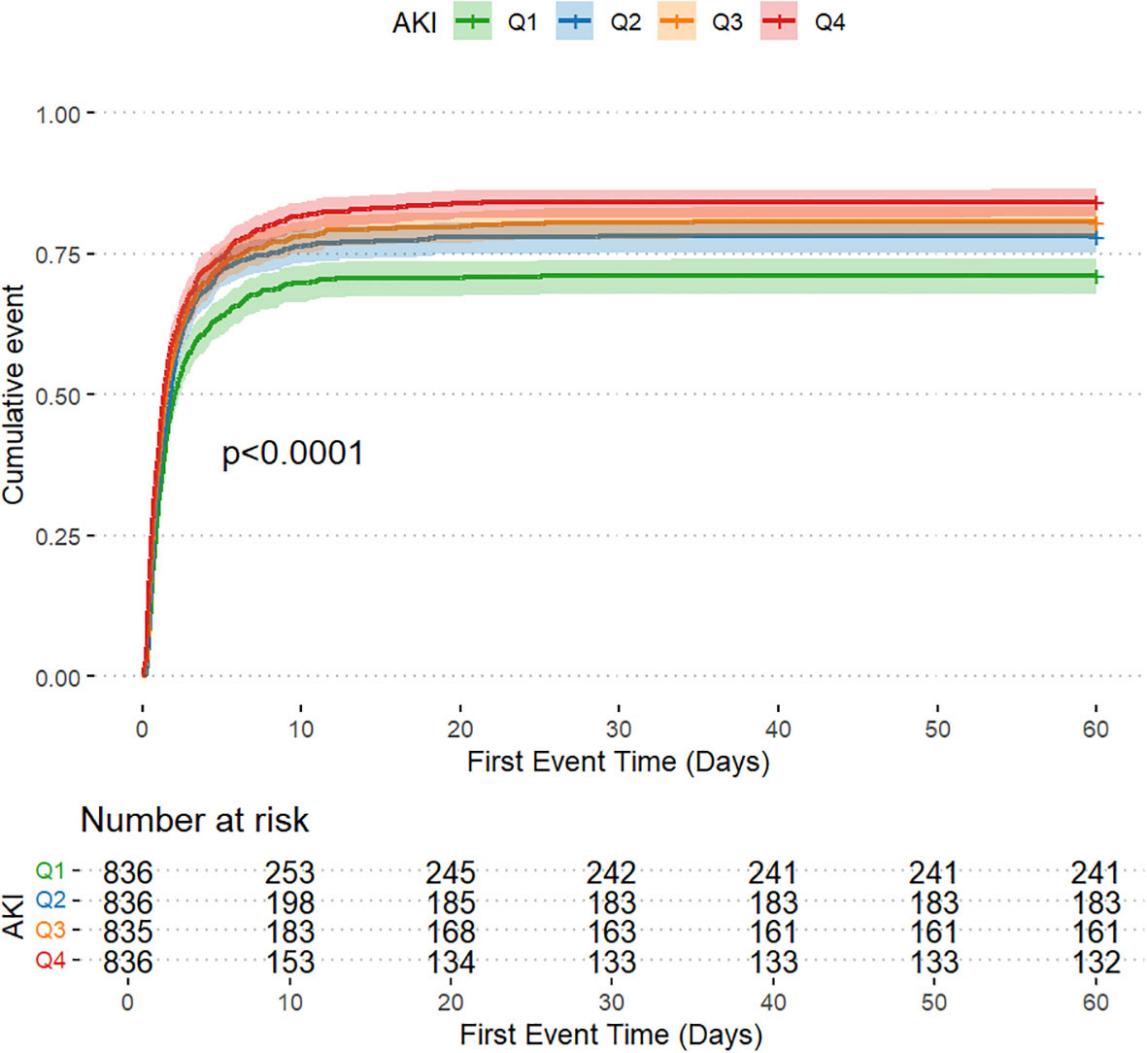
Cumulative event incidence curves for the incidence of acute kidney injury.

**Figure 4:**
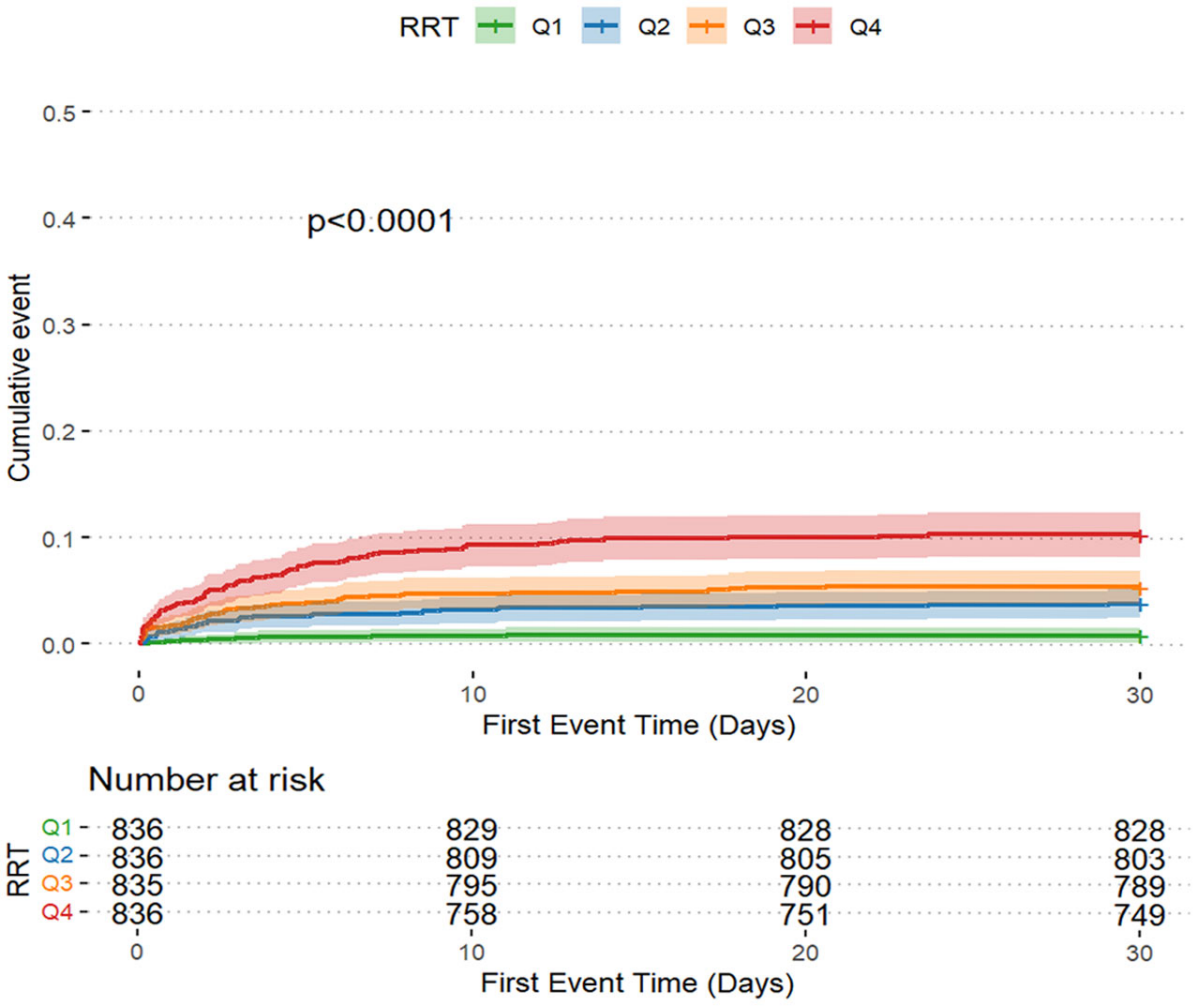
Cumulative event incidence curves for renal replacement therapy use.

The AKI incidence in the four groups of patients with CI were 71.2% (Q1), 78.1% (Q2), 80.7% (Q3) and 84.2% (Q4), underscoring the increased risk of AKI in Q4, with higher GV (**Table 2** and **Figure 2A**). The cumulative risk curve also indicated that AKI incidence in Q4 increased during the follow-up period (p<0.001) (**Figure 3**). RRT use and in-hospital mortality in Q4 were 10.4% and 21.3%, respectively, which were significantly higher than those in the other three groups, according to the stacked percentage bar chart (**Table 2** and **Figures 2B, C**). In addition, the cumulative risk curve indicated that RRT use in Q4 increased during the follow-up period (p<0.0001) (**Figure 4**).

The Cox regression model demonstrated that GV was independently associated with AKI incidence. When GV was treated as a categorical variable in the fully adjusted model 3, the risk of AKI for Q4, Q3, and Q2 were as follows: HR, 1.320 (95% CI: 1.133–1.537); HR, 1.299 (95% CI: 1.121–1.505); and HR, 1.166 (95% CI: 1.007–1.350), respectively. When GV was regarded as a continuous variable, the risk of AKI was HR, 1.349 (95% CI: 0.970–1.877) (**Table 1**). When GV was treated as a categorical variable in the fully adjusted model 3, the risks of RRT use for Q4, Q3, and Q2 were as follows: HR, 4.285 (95% CI: 1.789–10.261); HR, 2.609 (95% CI: 1.065–6.392); and HR, 2.222 (95% CI: 0.883–5.590), respectively. When the GV was regarded as a continuous variable, the risk of AKI was as follows: HR, 4.539 (95% CI: 1.753–11.752) (**Table 1**). Multivariate logistic regression analysis was used to assess the predictive risk of in-hospital mortality among patients with CI based on the GV values, and the fully adjusted model 3 yielded HRs of 1.135 (95% CI: 1.082–1.191); 1.054 (95% CI: 1.006–1.103); and 1.065 (95% CI: 1.018–1.114) for Q4, Q3, and Q2, respectively. When GV was treated as a categorical variable and the GV as a continuous variable, the HR was 1.326 (95% CI: 1.176–1.494) (**Table 1**). Moreover, after accounting for in-hospital mortality in the competing risk analysis, the incidence of AKI remained significantly different in the fourth group. In the fully adjusted Model 3, the hazard ratio (HR) was 1.66 [95% CI: 1.29–2.13], with a p-value < 0.001(**Supplementary Table S1**).

**Figure 5:**
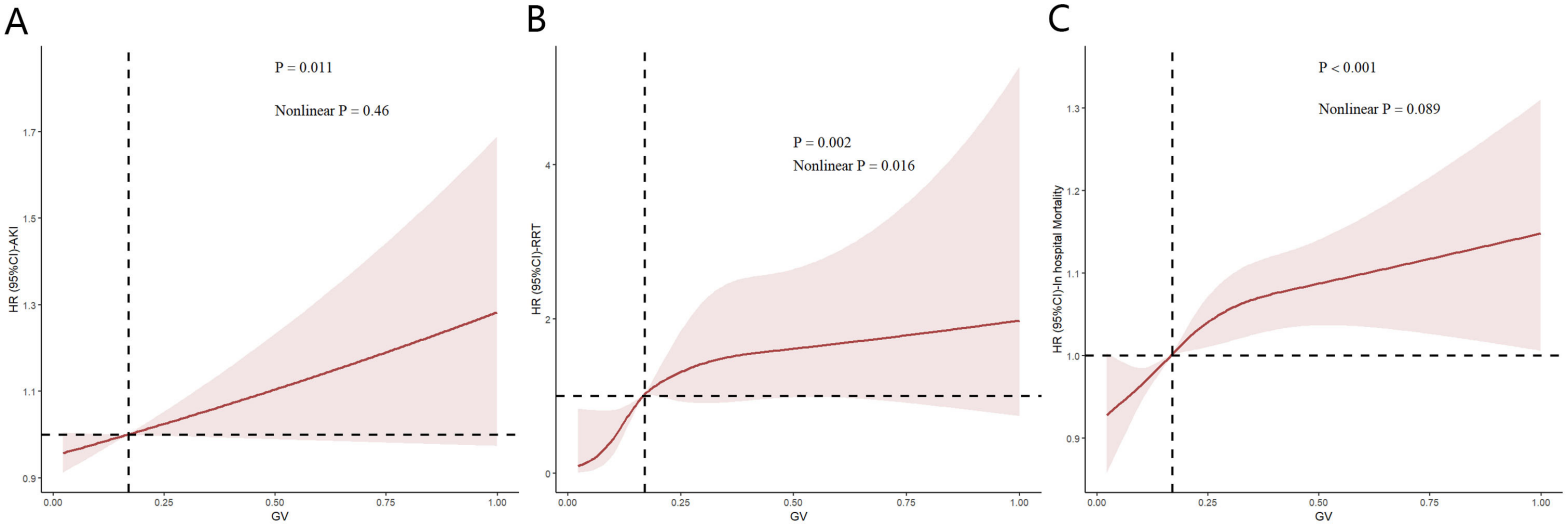
Restricted cubic spline (RCS) showing the relationship between glycemic variability (GV) and outcome indicators in the Medical Information Mart for the Intensive Care database. **(A–C)** RCS shows a correlation between GV and acute kidney injury incidence as well as in-hospital mortality in the fully adjusted Cox regression model. Nonlinear p value indicates whether GV had a linear correlation with the outcome, and p>0.05 indicates a linear correlation.

**Figure 5** illustrates the restricted cubic spline regression model based on the fully adjusted Cox regression of model 3, highlighting the dose-response relationship between GV and the risk of AKI (nonlinear p=0.46, p=0.011) and RRT use (nonlinear p=0.016, p=0.002). Restricted cubic spline regression models for in-hospital mortality demonstrated a relatively clear positive correlation with GC (nonlinear p=0.089 and p<0.001).

### Subgroup analyses

**Figure 6:**
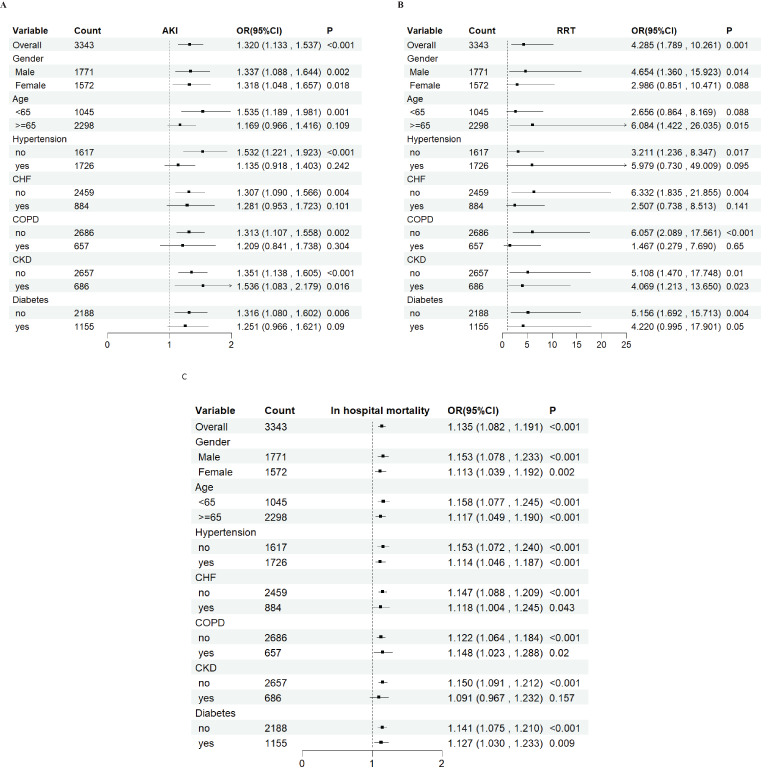
Subgroup analyses for the association of glycemic variability with acute kidney injury incidence, renal replacement therapy use, and in-hospital mortality. OR, odds ratio; CI, confidence interval.

To further examine the relationship of GV with AKI incidence, RRT use and in-hospital mortality, stratified analyses were performed based on sex (female vs. male), age (<65 vs. ≥65 years) and presence of conditions, such as HTN, CHF, COPD, CKD and DM. When the patients were stratified by sex (male = odds ratio [OR]: 1.337, 95% CI: 1.088–1.644; female = OR: 1.318, 95% CI: 1.048–1.657) and age (<65 years = OR: 1.535, 95% CI: 1.189–1.981; ≥65 years = OR: 1.169, 95% CI: 0.966–1.416), the fully adjusted model showed a significant relationship between GV and AKI incidence. Further, whether the patients were stratified by sex (male = OR: 4.654, 95% CI: 1.360–15.923; female = OR: 2.986, 95% CI: 0.851–10.471) or age (<65 years = OR: 2.656, 95% CI: 0.864–8.169; ≥65 years = OR: 6.084, 95% CI: 1.422–26.035), the fully adjusted model showed a significant relationship between GV and RRT use. Moreover, when stratified by sex (male = OR: 1.153, 95% CI: 1.078–1.233; female = OR: 1.113, 95% CI: 1.039–1.192) and age (<65 years = OR: 1.158, 95% CI: 1.077–1.245; ≥65 years = OR: 1.117, 95% CI: 1.049–1.190), the fully adjusted model showed a significant relationship between GV and in-hospital mortality. The same correlations were observed in patients with CHF, HTN, myocardial infarction, COPD, CKD and DM (**Figure 6**).

## Discussion

This study demonstrated that GV is a critical predictor of AKI incidence in patients with CI. Notably, this correlation remained significant in the MIMIC-IV patient cohort even after adjusting for potential confounding factors. Importantly, this study introduces a straightforward methodology for assessing the glucose control state to optimize the stratification of AKI risk in critically ill patients with CI.

Epidemiological surveillance data from 2020 demonstrate stroke prevalence rates of 2.6% (95%CI:2.6–2.6) in China, with corresponding incidence and mortality rates quantified at 505.2 (95%CI:488.5–522.0) and 343.4 (95%CI:329.6–357.0) per 100,000 person-years, respectively (13). Ischemic stroke (IS) constituted the predominant subtype, accounting for 87% of cases despite the conventional categorization of IS and hemorrhagic subtypes (14). CI is a major adverse event characterized by inadequate blood supply to the cerebral functional zone and is accompanied by reversible injury without apt reperfusion of the occlusion (15). AKI represents a clinically consequential complication in CI patients, mediated through multifactorial etiological pathways including hypovolemic states, contrast-induced nephrotoxicity, and baseline chronic comorbidities (16). A case-control study demonstrated lower AKI incidence (4.8% vs. 7.7%) among IS patients receiving computed tomography angiography (OR=0.66, 95%CI=0.35–1.22) compared to controls (17). CKD patients exhibit elevated susceptibility to AKI, with pre-existing CKD further amplifying AKI incidence in IS populations (18). Zhu et al. and Ma et al. found favorable concordance between AKI incidence and acute ischemic stroke (AIS) during ICU stay (19, 20).

Studies have shown that BG regulation disorders are correlated with a high risk of metabolic dysfunction-associated complications, such as cardiovascular, cerebrovascular, and kidney-vascular diseases (21, 22). Chi et al. elucidated the nephroprotective mechanisms of dapagliflozin in attenuating lipopolysaccharide-induced endotoxic shock and AKI-associated pathology in streptozotocin-induced diabetic murine models, mediated through adenosine monophosphate kinase pathway activation (23). Prior investigations have documented AKI as a prevalent complication among pediatric populations with newly diagnosed type 1 diabetes mellitus, exhibiting independent associations with elevated BG concentrations and diabetic ketoacidosis (DKA). Notably, all stage 3 AKI manifestations demonstrated exclusive correlations with DKA (24). The harmful effects of hyperglycemia on the kidneys are well known; however, the harmful effects of hypoglycemia cannot be underestimated. Hypoglycemia may lead to decreased renal blood flow and affect renal perfusion and function. Chronic hypoglycemia induces renal ischemic injury through pathophysiological mechanisms involving diminished microvascular perfusion, ultimately resulting in reduced glomerular filtration rate (25). In addition, hypoglycemia may affect the metabolic function of the kidney, leading to a decline in the ability of the kidney to regulate electrolyte and acid-base balance (26). Moreover, hypoglycemia may be related to an increase in renal injury markers, such as renal injury molecule-1 and neutrophil gelatinase-related lipoproteins (27). Controlling blood sugar over a wide range may not improve the long-term prognosis of patients; however, a narrow and accurate blood sugar control range and small variability interval need to be further explored.

This investigation acknowledges several methodological limitations. Primarily constrained by single-timepoint GV assessment at admission, the analysis lacks longitudinal glycemic profiling, precluding comprehensive evaluation of dynamic multi-organ dysfunction trajectories. Database limitations further restricted granular severity stratification for critical comorbidities (heart failure, hepatic insufficiency, malignancy). Moreover, unmeasured socioeconomic determinants may introduce residual confounding. And the study cohort predominantly comprised CI patients from the United States. Substantial differences in demographic characteristics—including racial/ethnic distribution, BMI profiles, insulin secretion patterns, and smoking prevalence—compared to Asian populations may limit the generalizability of the findings to other geographic or ethnic groups. External validation through multicenter prospective cohorts remains imperative to confirm these findings. And in our subgroup analysis, GV was not significantly associated with AKI among patients with pre-existing diabetes. One possible explanation is that patients with diabetes often exhibit long-term glycemic adaptations and may tolerate wider fluctuations in blood glucose levels due to chronic metabolic conditioning. In contrast, non-diabetic individuals may be more susceptible to glucose variability, which may trigger oxidative stress, endothelial dysfunction, or immune activation, thereby increasing the risk of AKI.

The results of this study show that GV, whether treated as a categorical or continuous variable, has a significant positive effect on the incidence of AKI, RRT use, and in-hospital mortality in patients with AIS, suggesting that higher GV values may synergistically enhance the damage caused by basic risk factors to the kidney.

## Conclusion

This analysis identified elevated GV as inversely associated with AKI incidence in CI patients. GV demonstrates independent prognostic utility for AKI risk stratification in this population and may inform targeted therapeutic strategies. Nevertheless, validation through large-scale prospective cohort studies and mechanistic investigations remains imperative.

## Data Availability

Publicly available datasets were analyzed in this study. This data can be found here: https://mimic.mit.edu/.

## Supplementary material

Supplementary Table 1 Competing risks model analysis. Model 1 was unadjusted. Model 2 was adjusted for sex and age. Model 3 was adjusted for age; sex; white blood cell and red blood cell counts; hemoglobin, serum creatinine, blood urea nitrogen, alanine aminotransferase, aspartate aminotransferase, sodium, calcium, and chloride levels; and the presence of chronic heart failure, hypertension, diabetes, and chronic kidney disease. The lowercase letter “a” following “quartile” indicates that, according to the research method described earlier, the two groups were divided based on the 25% quartile.
